# Isolation and Characterization of *Levilactobacillus brevis* FBL-3 from Godeulppaegi Kimchi and Screening of Medium Components for γ-Aminobutyric Acid Production

**DOI:** 10.4014/jmb.2608.08011

**Published:** 2026-09-22

**Authors:** Eun-Ju Jang, Veeranjaneya Reddy Lebaka, Young-Min Kim, Young-Jung Wee

**Affiliations:** 1 Department of Food Science and Technology, Yeungnam University, Gyeongsan 38541, Republic of Korea; 2 Department of Microbiology, Yogi Vemana University, Kadapa, AP 516005, India; 3 Department of Food Science & Technology, Chonnam National University, Gwangju 61186, Republic of Korea

**Keywords:** γ-Aminobutyric acid, *Levilactobacillus brevis*, Godeulppaegi kimchi, Glutamate decarboxylase, Medium optimization, L-Glutamic acid

## Abstract

*γ*-Aminobutyric acid (GABA) is an amino acid with a variety of biological functions and is now becoming an essential food ingredient. In this work, a GABA-producing lactic acid bacterium was isolated from godeulppaegi kimchi and characterized by phenotypic, biochemical and molecular parameters. The isolate was identified as FBL-3, which produced acid colonies in MRS agar containing CaCO_3_ and L-glutamic acid, was Gram positive, catalase-negative and rod-shaped, and had 99.1% similarity to *Levilactobacillus brevis* by API 50 CHL analysis and 100% 16S rRNA gene sequence similarity to *Lvb. brevis* ATCC 14869. Further, sequence analysis of 16S rRNA gene confirmed that it was *Lvb. brevis* FBL-3. The effects of carbon source, nitrogen source, and L-glutamic acid concentration on cell growth and TLC-densitometrically estimated GABA production were evaluated sequentially using a one-factor-at-a-time approach. Of the carbon sources tested, xylose had the highest GABA production (12.2 ± 2.34 g/L) and dry cell weight (0.75 ± 0.00 g/L) and 10 g/L xylose was selected as a good concentration for GABA production. Yeast extract was the most promising for nitrogen sources producing 4.65 ± 0.45 g/L GABA, and 20 g/L yeast extract was selected as a practical concentration since it produced 9.12 ± 1.48 g/L GABA with lower nitrogen input than 50 g/L yeast extract. L-Glutamic acid was a necessary precursor to GABA production. Although the highest GABA titer in the L-glutamic acid concentration experiment was found for 50 g/L L-glutamic acid (19.6 ± 1.03 g/L), 10 g/L L-glutamic acid supported the highest cell growth and was considered as a more balanced precursor.

## Introduction

γ-Aminobutyric acid (GABA) is a four-carbon non-protein amino acid that serves as one of the major inhibitory neurotransmitters in the mammalian central nervous system, and is also present in plants and animals, and in microorganisms [1, 2]. Besides the neurological aspect of the substance, GABA has also received considerable attention as a bioactive food element, with recent clinical and review studies showing that oral GABA is associated with reduced perceived stress, better sleep quality, and blood pressure lowering, at low levels, and can be well tolerated. However, the natural GABA content of most foods is low, and fermentation is considered to be the best way to create GABA-containing food products [6]

Biologically, GABA is produced from L-glutamate with a single decarboxylation process catalyzed by glutamate decarboxylase (GAD; EC 4.1.1.15) with pyridoxal-5′-phosphate as its cofactor [7, 8] in lactic acid bacteria (LAB). Lactic acid bacteria (LAB) have been linked to the glutamate decarboxylase system which has intracellular GAD enzymes and the glutamate/GABA antiporter GadC. The glutamate decarboxylase system takes up protons from the intracellular environment as a substrate for glutamate decarboxylation, and therefore can result in acid resistance and GABA production [8-11].

LAB isolated from fermented foods are an attractive microbial site of GABA production since many of them are known to be safe for food fermentation. Korean fermented foods such as kimchi, jeotgal, and fermented soybean products are often reported as sources of GABA-producing LAB [12-15] and have been proven to be reliable sources of GABA production in the laboratory. *Levilactobacillus brevis* has been studied extensively among LAB because many strains have active GAD systems and can convert exogenous MSG (L-glutamic acid) into GABA [9, 10, 16-19].

Although several *Lvb. brevis* strains have been reported, GABA productivity depends on strain origin, GAD activity, carbon source, nitrogen source, MSG concentration, initial pH and fermentation strategy [14, 20-22]. Thus, medium optimization is important for each newly isolated strain. Carbon sources influence energy production and redox balance, while nitrogen sources provide amino acids, peptides, vitamins and growth factors to create biomass and enzyme activity. L-Glutamic acid is the immediate precursor to GABA biosynthesis but high precursor levels can change osmotic pressure, pH behavior, and conversion efficiency [20, 22, 23]. Genetic and enzymatic engineering methods, such as reconstruction of the GAD system in heterologous hosts or design of consensus GAD enzymes, have been explored to increase GABA yield beyond the medium optimization alone, showing the need to identify new native GABA-producing strains as starting biocatalysts [24, 25].

Accordingly, the aim of this study was to isolate and identify a GABA-producing LAB strain from godeulppaegi (Korean bitter lettuce) kimchi and to screen selected medium components and concentrations as a preliminary basis for subsequent multivariable process optimization. The isolated strain was identified as *Lvb. brevis* FBL-3, and we investigated its medium requirements to further study food-grade GABA fermentation.

## Materials and Methods

### Isolation of GABA-Producing Bacteria

Cabbage kimchi, godeulppaegi kimchi, young radish kimchi, and dongchimi (Korean watery kimchi) samples were taken from home or purchased. A 1 mL aliquot of each kimchi suspension was inoculated into *Lactobacillus* MRS broth (MRS; Difco Laboratories, USA) with 1% (w/v) L-glutamic acid and incubated at 37°C for 24 to 48 h. Cultures were diluted with sterile saline solution and spread over MRS agar plates with 1% (w/v) CaCO_3_ and 1% (w/v) L-glutamic acid and incubated at 37°C for 48 h. The clear zones of acid production were selected and GABA production was detected by thin-layer chromatography (TLC) for selected isolates preserved at -80°C in culture broth containing glycerol at a 1:1 (v/v) ratio.

### Media and Culture Conditions

The preculture medium was MRS broth with 10 g/L L-glutamic acid. A frozen stock culture (1 mL) was inoculated in 39 mL of preculture medium in a 50 mL vial and incubated anaerobically at 37°C for 24 h in a shaking incubator (VS-8480SF; Vision Scientific Co., Republic of Korea). Unless otherwise stated, 1 mL of the preculture was transferred into 39 mL of production medium containing K_2_HPO_4_ (1 g/L), the carbon source, the nitrogen source, and the concentration of L-glutamic acid. Cultures were incubated anaerobically at 37°C and 200 rpm for 48 h in a shaking incubator. The initial pH of the production medium was adjusted to 6.2 prior to inoculation. No additional pH-control step was applied during the 48-h production cultures, and the final culture broth reached a pH of 4.5.

### Morphological and Cultural Characterization

The selected isolate was characterized through cultural and cellular morphological properties. For cultural characterization, the isolate was streaked on MRS agar and GABA-production agar with L-glutamic acid and the plates were anaerobically incubated at 37°C for 48 h. After incubation, colony morphology was studied by measuring colony size, shape, elevation, margin, surface texture, opacity, pigmentation, and the presence of clear zones around colonies. The presence of clear zones on CaCO_3_-containing agar was viewed as an indicator of acid production which is a typical characteristic of lactic acid bacteria.

Cell morphology was investigated by scanning electron microscopy (SEM) with an S-4100 microscope (Hitachi Co., Japan). Briefly, after 24 h of cultivation in MRS broth, bacterial cells were centrifuged and washed twice with sterile distilled water to remove residual medium components and impurities. The washed cell pellets were fixed with 2.5% glutaraldehyde to preserve cellular structure and then post-fixed with 1% osmium tetroxide to improve membrane contrast and structural stability. The cells were dehydrated through a graded ethanol series, dried with a critical-point dryer (HCP-2; Hitachi) and then coated with a thin conductive layer with a precision etching coating (Gatan 682; Gatan, USA). The sample was examined microscopically to determine cell shape, arrangement, surface characteristics and approximate cell size of the isolate.

### Physiological and Biochemical Characterization

The physiological and biochemical properties of the chosen isolate were assessed by standard microbiological tests. Gram staining was performed to identify the cell wall characteristics and the characteristics of the isolate as Gram-positive or Gram-negative. The activity of the catalase was examined by applying hydrogen peroxide solution to the newly grown bacterial cells and the formation of oxygen bubbles was observed. The absence of bubble formation could be regarded as a catalase-negative reaction in lactic acid bacteria.

Organic acid production was also determined by using CaCO_3_-based agar medium. The isolate was streaked onto agar plates with CaCO_3_ and incubated at 37°C for 48 h. The formation of a clear zone around the colony was observed as a positive sign that the colony was solubilized with CaCO_3_. This is the result of acid production by the bacteria which dissolves insoluble calcium carbonate in the surrounding medium.

The carbohydrate utilization profiles were assessed using the API 50 CHL system (bioMérieux, France). Briefly, bacterial cells were collected from a growing culture, suspended in API 50 CHL medium, and inoculated into the microtubes containing the individual carbohydrate substrates. The strips were incubated at 37°C and the color of the strips was monitored for any positive or negative carbohydrate fermentation reactions. The resulting biochemical profile was compared to the API 50 CHL database version 5.2 for species-level identification of the isolate. These phenotypic and biochemical results were combined with molecular identification to confirm the taxonomic property of the selected GABA-producing strain.

### 16S rRNA Gene Sequencing and Phylogenetic Analysis

Molecular analysis of the isolate was carried out by sequencing the 16S rRNA gene. Bacterial cells were taken from a 24 h culture by centrifugation at 15,000 rpm for 10 min and used for genomic DNA preparation. DNA was extracted using boiling-based extraction with Chelex 100 resin (Bio-Rad Laboratories, USA) and then purified to remove PCR-inhibitory components and prepare DNA suitable for amplification. The 16S rRNA gene fragment was amplified using polymerase chain reaction with bacterial universal primer pair 27F (5′-AGAGTTTGAT CCTGGCTCAG-3′) and 1492R (5′-GGCTACCTTGTTACGACTT-3′). The amplified PCR products were purified to remove any remaining primers, nucleotides, enzymes, and buffer components before sequencing. We then performed nucleotide sequencing using the BigDye Terminator v3.1 Cycle Sequencing Kit, and generated the sequence data with an ABI 3730XL DNA Analyzer (Thermo Fisher Scientific, USA).

The 16S rRNA gene sequence was then edited and compared to reference sequences available in public taxonomic databases. We searched the sequences with the Blastn in the National Center for Biotechnology Information (NCBI) database and EzTaxon database to find bacteria that are similar in DNA sequence. The sequence of the isolate was aligned with those of related reference strains using Clustal X and the alignment was checked and improved with BioEdit. We then constructed the phylogenetic tree using the neighbor-joining method in MEGA to measure the evolution of the isolate from the closest taxonomic relatives. The database comparison and phylogenetic clustering were combined to confirm the taxonomic identity of the selected GABA-producing strain.

### Effect of Medium Components on Cell Growth and GABA Production

The effect of the medium composition on bacterial growth and GABA biosynthesis was investigated by modifying one nutritional factor on the basis of the remaining components and leaving the rest of the nutritional factors. To determine a suitable carbon source, the various production media were separately supplemented with different carbon sources. Yeast extract, L-glutamic acid, and K_2_HPO_4_ were kept at 10 g/L, 10 g/L, and 1 g/L respectively. After cultivation, cell growth and GABA accumulation were compared among the carbon sources. The carbon source that produced the highest GABA levels was then selected to further optimize the carbon source, and the concentration of the carbon source was adjusted in a range from 0−50 g/L to assess the concentration-dependent response of the isolate.

Eight nitrogen-containing ingredients or compounds – yeast extract, beef extract, malt extract, peptone, tryptone, urea, corn steep liquor, and ammonium chloride – were screened separately at the same ingredient concentration (10 g/L). This fixed-mass design was intended as a practical ingredient-level comparison and not as an equivalent nitrogen experiment. Because the tested materials differ in total nitrogen, peptide and amino acid composition, vitamins, minerals, and other growth factors, the results cannot be interpreted as the effect of nitrogen amount alone. During this screening step, the medium was filled with xylose at 10 g/L, L-glutamic acid at 10 g/L, and K_2_HPO_4_ at 1 g/L. After incubation, cell growth and GABA production were measured, and the nitrogen source which had the most effect in increasing GABA was chosen for concentration optimization. The nitrogen source was further used in a range of 0 to 50 g/L to determine the appropriate concentration which would be effective for GABA formation without over-supplying the medium.

Because GABA is produced by decarboxylation of L-glutamate, the effect of precursor availability was also investigated. L-Glutamic acid was added to the production medium at 0−50 g/L, and the selected carbon and nitrogen sources were kept at their optimal concentrations. This experiment was conducted in order to determine whether GABA production from the isolate was limited by precursor concentration and how substrate availability, cell growth, and final GABA accumulation were related to substrate availability. All cultures were incubated anaerobically at 37°C and 200 rpm for 48 h, and cell biomass and GABA content in culture broths were measured.

### Analytical Methods

Cell growth was quantified as dry cell weight (DCW) to provide a biomass-based index of the growth of the selected isolate across different cultures. At the end of each cultivation period, culture samples were collected and diluted with sterile distilled water. The optical density of each diluted sample was measured at 660 nm using a UV-1201 spectrophotometer (Shimadzu Co., Japan). If the optical density exceeded 0.7, further dilution was performed to minimize errors from excessive light scattering. To convert optical density to DCW, a calibration curve was prepared using bacterial cultures grown under identical conditions. Small volumes of culture broth were centrifuged to collect cell pellets, which were subsequently washed to remove residual culture broth and freeze-dried until a constant dry mass was achieved. The dry biomass values were plotted against optical density values to establish a linear relationship between optical density and cell concentration. The DCW of experimental samples was calculated from this calibration equation and expressed as grams of dry biomass per liter of culture broth.

The concentrations of GABA and residual L-glutamic acid were measured by TLC coupled with densitometric image analysis. Although this approach is useful for rapid comparison of multiple fermentation samples, the values obtained in this study should be interpreted as TLC-densitometry-based estimates. After cultivation, culture samples were centrifuged at 9,000 rpm for 10 minutes to remove bacterial cells and insoluble particles. The supernatant was collected and used for analysis. For each analysis, 1 μL of the supernatant was applied to silica gel 60 F254 TLC plates (Merck, Darmstadt, Germany), along with standards of GABA and L-glutamic acid prepared at known concentrations. The TLC plates were developed in a solvent system of n-butanol, acetic acid, and water (5:3:2 v/v/v). To enhance separation of GABA and L-glutamic acid spots, the development process was repeated three times under identical solvent conditions. After development, the plates were dried at room temperature to remove residual solvent. Spots were visualized by spraying with a 0.2% ninhydrin solution prepared in ethanol and heating at 110°C for 10 min. Under these conditions, GABA and L-glutamic acid appeared as ninhydrin-positive spots on the TLC plate.

The color intensity of the sample spots was quantified using standard solutions. Spot areas and intensities were measured using NIH Image software, and calibration curves for standard GABA and L-glutamic acid solutions were generated. The concentrations of GABA and residual L-glutamic acid in the culture supernatants were calculated from the corresponding standard curves. This method was utilized to monitor both GABA accumulation and precursor consumption during medium optimization experiments.

Average volumetric productivity was calculated from the endpoint measurement as P_GABA_ = C_GABA_/48 h. For the highest-titer precursor condition, apparent molar conversion relative to supplemented L-glutamic acid was calculated as Y_app_ = [(C_GABA_/103.12)/(C_L-glutamic acid_/147.13)] × 100, where 103.12 and 147.13 g/mol are the molecular weights of GABA and L-glutamic acid, respectively. This apparent value does not correct for glutamate contributed by complex medium components or inoculum carryover and is not equivalent to a yield based on measured substrate consumption.

### Statistical Analysis

All the experimental data were obtained in three independent replicates and the results are presented as the mean value with standard deviation. We compared the treatment groups to determine whether the change in medium composition caused significant differences in cell growth or GABA production. The data were first analyzed by one-way analysis of variance using SPSS software version 23.0 (SPSS Inc., USA). If the treatment effect was significant, Duncan’s multiple range test was performed to separate the mean. Differences were considered statistically significant if *p* < 0.05.

## Results and Discussion

### Isolation and Screening of GABA-Producing Bacteria

To isolate GABA-producing lactic acid bacteria, different kimchi samples were collected from household and local markets in kimchi (cabbage kimchi, godeulppaegi kimchi, young radish kimchi, dongchimi). Each kimchi sample was suspended and 1 mL of the suspension was inoculated into sterile MRS broth and incubated for lactic acid bacteria enrichment. Those cultures were diluted serially and placed on agar. On incubation, a few colonies with acid production activity were identified based on their formation and the presence of clear zones on the screening medium.

The selected colonies were inoculated in MRS broth with 1% L-glutamic acid, and statically incubated at 37°C for 24 h. The culture supernatants were then analyzed by thin-layer chromatography to determine GABA production (Fig. 1). Among all isolates studied, the strain with a rather strong and distinct GABA spot (K5 in Fig. 1D) was found to be the final candidate for further characterization. This isolate was labeled FBL-3 and used for identification and medium optimization experiments.

TLC- or HPTLC-based densitometric methods have been applied for GABA quantification in biological and food-related samples, and a previous study on *Lvb. brevis* reported a good correlation between TLC image analysis and RP-HPLC for GABA detection [26-28]. Nevertheless, because HPLC confirmation was not performed in the present study, further validation using HPLC-based amino acid analysis will be necessary to confirm the absolute GABA concentrations and conversion yields under the selected culture conditions.

Several LAB species associated with kimchi and other fermented foods, such as *Lentilactobacillus buchneri*, *Lvb. brevis* and its closely related species are known to produce GABA from glutamate [12, 14, 15, 29]. The selection of FBL-3 in godeulppaegi kimchi is thus consistent with the microbial ecology of fermented vegetables, where acid-tolerant LAB with amino acid conversion ability could be maintained in the fermentation phase. A more detailed comparison of the isolation frequency and relative abundance of GABA-producing LAB among the total culturable population of each kimchi type (*e.g.* colony count-based enrichment ratios or amplicon-based community profiling for other kimchi microbiomes [34-36]) may help determine how representative FBL-3 was among the LAB populations recovered from godeulppaegi kimchi samples and is therefore a direction worth further study.

### Morphological, Cultural, Physiological, and Biochemical Characterization of Strain FBL-3

The cultural traits of strain FBL-3 were analyzed on both MRS agar and GABA-production agar (Fig. 2). On MRS agar the strain FBL-3 formed circular milky white colonies with a convex center and a relatively large colony size. Colonies grown on GABA-production agar were similar in color and general shape to those on MRS agar, but the size of colonies was smaller, suggesting that the composition of GABA-production medium could affect colony development or growth rate in the solid-state culture.

Cell morphology was also measured with scanning electron microscopy. As shown in Fig. 3, strain FBL-3 appeared as the single, rod-shaped cells with a length of 1.5−2.0 μm. The microscopic structure of the cells was in line with the general microscopic character of rod-shaped lactic acid bacteria. Gram staining confirmed the isolate was Gram-positive rod and the catalase test showed a negative reaction. Moreover, strain FBL-3 produced a clear zone on CaCO_3_-containing agar, implying that acid is being produced during the growth process. These phenotypic features such as Gram-positive staining, catalase negativity, rod-shaped morphology, and acid formation support the classification of strain FBL-3 as a lactic acid bacterium.

API 50 CHL test was carried out to better characterize the biochemical profile of strain FBL-3 and the results are shown in Table 1. The isolate showed positive response for L-arabinose, ribose, D-xylose, D-fructose, maltose, and 5-keto-gluconate and negative for esculin hydrolysis, associated with β-glucosidase activity. It was reported that *Lvb. brevis* KCCM 40399 uses L-arabinose, ribose, D-xylose, D-galactose, D-glucose, N-acetyl-glucosamine, D-fructose, maltose, and gluconate, and *Lvb. brevis* KCTC 3498 shows positive reactions for L-arabinose, ribose, D-xylose, D-mannose, and maltose [31, 32].

Although the carbohydrate assimilation profile of strain FBL-3 was not identical to that of the other comparison strains, several positive reactions (especially for L-arabinose, ribose, D-xylose, D-fructose, and maltose) were present with the biochemical characteristics of *Lvb. brevis* strains, formerly classified as *Lactobacillus brevis*. From the API 50 CHL database analysis, strain FBL-3 was found to have 99.1% similarity to *Lvb. brevis*. This result indicated that the isolate belonged to *Lvb. brevis* species group. However, phenotypic and biochemical identification systems could be affected by strain level variations, culture conditions, and carbohydrate metabolism of closely related lactic acid bacteria. Thus, the API 50 CHL result alone was not sufficient to determine the classification of the isolate.

### 16S rRNA Gene Sequence Analysis and Phylogenetic Identification of Strain FBL-3

To enhance the reliability of taxonomic identification, we amplified and sequenced the 16S rRNA gene from strain FBL-3. The resulting sequence was deposited in the NCBI GenBank under the accession number PZ746795. Using the Blastn tool within the NCBI database for comparison, we discovered that strain FBL-3 is closely related to the *Lactobacillus* genus. As indicated in Table 2, the 16S rRNA gene sequence of strain FBL-3 exhibits over 98% similarity to several strains of lactic acid bacteria, including *Lvb. brevis* ATCC 14869, *Lvb. hammesii* TMW 1.1236, *Lvb. yonginensis* THK-V8, and *Lvb. senmaizukei* L13. Among these related taxa, the closest match was with *Lvb. brevis* ATCC 14869, which showed 100% sequence similarity.

Based on these sequence comparison findings, we conducted multiple sequence alignments between the 16S rRNA gene of strain FBL-3 and reference strains with over 94% similarity. As illustrated in Fig. 4, strain FBL-3 is very closely related to the *Lvb. brevis* ATCC 14869 species group, thereby reinforcing the taxonomic connection of this strain to *Lvb. brevis*. Through analysis of the API 50 CHL profile, the 16S rRNA gene sequence similarity, and the phylogenetic relationship, we classified the isolate as *Lvb. brevis* FBL-3 for further experimentation. Following the reclassification of Lactobacillus in 2020, the bacterial name is now *Levilactobacillus brevis* [17].[Fig F5]

The close relationship between strain FBL-3 and *Lvb. brevis* ATCC 14869 is notable from a metabolic perspective. For instance, *Lvb. brevis* ATCC 14869, when grown for 22 h in MRS medium supplemented with glucose and xylose, consumed 11.1 g/L of glucose and 10.5 g/L of xylose, yielding 12.5 g/L of lactate, 4.5 g/L of acetate, and 0.7 g/L of ethanol [33]. These fermentation characteristics suggest that *Lvb. brevis* ATCC 14869 can effectively utilize both hexose and pentose sugars while producing organic acids through heterofermentative metabolism. In this work, *Lvb. brevis* FBL-3 demonstrated 100% 16S rRNA gene sequence similarity with *Lvb. brevis* ATCC 14869 and also exhibited the ability to utilize xylose, indicating that both strains may share similar carbohydrate metabolic capabilities.

### Effect of Carbon Sources on Cell Growth and GABA Production

The effect of carbon source in the growth of *Lvb. brevis* FBL-3 and the GABA production ability was probed by adding eight different carbon sources to the production medium. Carbon availability is important in microbial fermentation as it provides metabolic energy for cell growth and precursor conversion in GABA production. After cultivation, dry cell weight and GABA concentration were compared to determine the suitability of each carbon source. Fructose, xylose, maltose, and glucose supported relatively high GABA production compared with the other carbon sources. The GABA concentrations obtained with fructose, xylose, maltose, and glucose are 12.0 ± 2.29, 12.2 ± 2.34, 11.57 ± 2.11, and 11.87 ± 2.11 g/L, respectively. Cell growth also differed on the carbon source. The dry cell weights obtained with xylose, maltose, fructose, and glucose are 0.75 ± 0.00, 0.73 ± 0.00, 0.58 ± 0.02, and 0.31 ± 0.03 g/L, respectively. These results suggest that xylose is the most favorable carbon source for biomass formation and GABA accumulation under the conditions tested. Even though xylose provided the highest GABA production, the difference between xylose and fructose is not much. This suggests that *Lvb. brevis* FBL-3 can use xylose, a pentose sugar, as fermentable carbon sources as well as fructose, a hexose sugar. Despite lactose, starch, and sucrose being fermentable carbon sources, cell growth and GABA production were similar to that in the medium but without added carbon sources. These results suggest that *Lvb. brevis* FBL-3 cannot metabolize these substrates in the present culture.

The property that *Lvb. brevis* FBL-3 can use glucose and xylose is consistent with the metabolic behavior of heterofermentative lactic acid bacteria. Generally, in lactic acid bacteria, carbohydrate catabolism takes place *via* glycolysis or the pentose phosphoketolase pathway. Recent physiological and genomic studies of Lactobacillaceae show that xylose is imported by a separate xylose isomerase/xylulokinase system and transferred into the phosphoketolase pathway, where it is converted to xylulose-5-phosphate and then acetyl-phosphate and glyceraldehyde-3-phosphate, giving rise to lactate and acetate, ethanol, and CO_2_ as typical heterofermentation end products [11, 17, 34, 35]. Homofermentative LAB primarily catabolize hexoses by glycolysis and give rise to lactate as the main end product. On the other hand, heterofermentative LAB metabolism of hexoses and pentoses by the phosphoketolase pathway results in lactate as well as acetate, ethanol, and carbon dioxide [35, 36]. Hence, the utilization pattern found in *Lvb. brevis* FBL-3 indicates that this isolate is heterofermentative in metabolism.

The GABA-producing ability of *Lvb. brevis* FBL-3 is also different from the GABA-producing lactic acid bacteria previously found in Korean fermented foods. For example, *Lvb. brevis* OPK 3 and *L. buchneri* from traditional fermented foods are reported to produce 19 mM and 251 mM GABA, respectively [7]. More recently, *Lvb. brevis* GABA100 and engineered *Lactococcus lactis* expressing heterologous GAD have also been reported to reach GABA titers > 100 g/L under fed-batch or substrate-feeding conditions, demonstrating the wide range of GABA productivity that can be achieved *via* strain selection and process design [13,25]. Although direct comparison among studies should be done in order to be sensitive to differences in culture conditions, media composition, precursor concentration, and analytical methods, the GABA level obtained from strain FBL-3 indicates that this isolate has good potential to produce GABA as a lactic acid bacterium.

If only GABA production and biomass creation were considered, xylose is better than the other carbon sources. However, glucose seems to produce a relatively high amount of GABA with respect to cell biomass produced, and as such, it could be better from the production efficiency perspective per unit of biomass. Nevertheless, xylose was the best carbon source in this study as it produced the highest amount of GABA and supported the greatest cell growth under the experimental conditions. These results suggest that xylose is a carbon source that helps to facilitate growth and GABA biosynthesis in *Lvb. brevis* FBL-3.

Since xylose is the best carbon source for cell growth as well as GABA production, we investigated the effect of xylose concentration from 0 to 50 g/L. The changes in biomass formation and GABA accumulation are shown in Fig. 6. The cell growth was highest when xylose was supplied at 10 g/L and the dry cell weight was 0.66 ± 0.01 g/L. However, increasing the xylose concentration did not increase biomass production. Cell growth gradually decreased as the xylose concentration increased above 10 g/L. The same trend was observed in GABA production. The highest concentration of GABA, 15.3 ± 1.14 g/L, was obtained at 10 g/L xylose, and as xylose concentration increased, the GABA accumulation gradually decreased.

The results that we obtained show that xylose is relevant for the growth and GABA production of *Lvb. brevis* FBL-3, but excessive supplementation is not beneficial. The decrease in GABA production at xylose concentrations above 10 g/L is probably due to substrate inhibition or osmotic pressure and/or the imbalance between carbon production and precursor conversion. In lactic acid bacteria, an excess of carbon source does not always lead to higher metabolite production because the cell may be focused on stress adaptation or maintenance metabolism or acid tolerance rather than on the production of the target products [8, 10]. This is similar to what happened in xylose-fed *Lvb. brevis* fermentations, where activation of the *xyl* operon increased GABA titer up to an optimal xylose feed level, beyond which no further substrate addition was of any benefit and could even decrease conversion efficiency [36]. Therefore, the decrease in both biomass and GABA production at higher xylose levels suggests that *Lvb. brevis* FBL-3 requires an appropriate carbon supply and not an excessive amount of fermentable sugar.

Although xylose was essential for efficient GABA biosynthesis, it was clear that the optimum concentration was 10 g/L in the culture at this time. *Lvb. brevis* FBL-3 showed the highest GABA production with favorable cell growth at this concentration. Hence, we chose 10 g/L xylose as the optimal carbon source concentration for subsequent medium optimization experiments.

### Effect of Nitrogen Sources on Cell Growth and GABA Production

We have evaluated the influence of nitrogen sources on *Lvb. brevis* FBL-3 growth and GABA production. Nitrogen is crucial for biomass production and cellular metabolism, enzyme synthesis, and amino acid conversion. Therefore, eight nitrogen sources were added to the production medium individually, and their effects on dry cell weight and GABA accumulation were compared.

As shown in Fig. 7, the type of nitrogen source also played a significant role in cell growth and GABA production. Yeast extract and beef extract showed much higher GABA production rates than the other nitrogen sources. The GABA concentrations with beef extract and yeast extract were 2.67 ± 0.19 and 4.65 ± 0.45 g/L, respectively. The nitrogen supplements also led to cell growth, with dry cell weights of 0.61 ± 0.00 g/L for beef extract and 0.72 ± 0.01 g/L for yeast extract. Yeast extract produced the highest biomass formation and GABA production, and thus it is the best nitrogen source for *Lvb. brevis* FBL-3.

The good effects of yeast extract can be attributed to its rich nutritional composition. Yeast extract provides readily available amino acids, peptides, vitamins, nucleotides, and other growth-promoting factors that can support LAB metabolism. Vitamin B_6_ (pyridoxal-5′-phosphate) is a key cofactor for glutamate decarboxylase activity, and yeast extract is a relatively rich natural source of this vitamin, so it may be the reason why it performs better than simpler organic nitrogen sources in facilitating GABA conversion [8, 25]. These compounds can improve cellular function and may be crucial cofactors or precursor-related nutrients to the glutamate conversion process. Corn steep liquor and ammonium chloride were not able to improve GABA production or biomass formation compared to the nitrogen-free control medium. In some cases, the effects were comparable to or lower than those of the control medium, suggesting that these nitrogen sources were not exploited by *Lvb. brevis* FBL-3 efficiently or were not sufficient to support the metabolic conditions for GABA biosynthesis.

At the fixed concentration of 10 g/L, yeast extract produced the highest biomass and GABA concentration, while beef extract showed the second-highest response. Peptone and tryptone supported intermediate biomass formation but yielded much less GABA than yeast extract or beef extract. Malt extract provided little improvement, and urea, corn steep liquor, and ammonium chloride did not support the same combination of growth and GABA accumulation observed with yeast extract. These differences may reflect not only total nitrogen but also the availability of peptides and amino acids, vitamins, minerals, and cofactors that support cellular metabolism and glutamate decarboxylase activity. In particular, the favorable response to yeast extract should be interpreted as an ingredient-specific effect under equal mass supplementation, not as evidence that it is superior on an equal-nitrogen basis. Future work should measure total nitrogen and compare the sources at equivalent nitrogen levels to separate nitrogen quantity from compositional effects.

Previous studies have shown that GABA-producing microorganisms isolated from fermented foods or plant-based materials differ considerably in their production capacity. *Lvb. brevis* K203 isolated from kimchi produced 44.4 g/L GABA, *Lvb. brevis* CE701 isolated from pickles produced 40.35 g/L GABA, and *Lactiplantibacillus plantarum* K154 isolated from kimchi produced 154.86 μg/mL GABA [11, 21, 36]. Other studies reported that *Bacillus subtilis* B060 from rice straw produced 20.58 mg/mL GABA, and *Lvb. brevis* CRL 1942 from quinoa sourdough produced 255 mM GABA [12, 22]. These studies show that GABA productivity is highly strain-dependent and depends very much on medium composition, precursor concentration, and fermentation conditions.

Although the experimental conditions and units of measurement are different between these studies, the results are very similar, and the findings from *Lvb. brevis* CRL 1942 are quite similar to what is also found in the previous studies. *Lvb. brevis* FBL-3 is capable of producing GABA and is sensitive to the nitrogen content of the medium. The increased GABA production in the yeast extract-supplemented medium suggests that the nutritionally rich organic nitrogen source is beneficial for cell development and GABA biosynthesis in *Lvb. brevis* FBL-3.

Yeast extract was added to the production medium at concentrations ranging from 0 to 50 g/L, and the changes in dry cell weight and GABA level are shown in Fig. 8, and the nitrogen content in the medium was optimal for the medium optimization experiments. The cell growth was most favorable with a yeast extract concentration of 10 g/L, and the dry cell weight was 0.66 ± 0.00 g/L. This indicates that yeast extract was required to support the active growth of *Lvb. brevis* FBL-3, probably because it contains many complex nutrients such as amino acids, peptides, vitamins, and other growth factors. Increasing the yeast extract concentration above this level did not necessarily lead to the corresponding increase in cell growth, suggesting that the nitrogen requirement for biomass production could have been adequately met at a relatively moderate concentration.

Mean biomass increased at the higher yeast-extract concentrations, with the highest dry cell weight observed at 50 g/L. GABA production increased sharply when yeast extract was added and then formed a broad response plateau: 20 g/L and 50 g/L yielded 9.12 ± 1.48 and 9.34 ± 0.51 g/L GABA, respectively, and the 10–50 g/L treatments were not significantly different in Fig. 8B. Thus, 50 g/L gave the highest mean GABA titer, whereas 20 g/L was selected as a practical working concentration for the subsequent precursor experiment because it achieved a comparable mean titer with 60% less yeast extract. This choice is an input-efficiency rationale, not a demonstrated economic optimum, because medium cost was not formally analyzed.

### Effect of L-Glutamic Acid Concentration on Cell Growth and GABA Production

The L-glutamic acid was used as a natural precursor to GABA biosynthesis and was added to the production medium at concentrations ranging from 0 to 50 g/L. The changes in cell growth and GABA accumulation are depicted in Fig. 9. No single L-glutamic acid concentration maximized both biomass and GABA accumulation. The 10 g/L treatment supported the highest dry cell weight (0.68 ± 0.05 g/L), whereas the 50 g/L treatment produced the highest TLC-densitometrically estimated GABA concentration (19.6 ± 1.03 g/L). Therefore, 10 g/L is described as growth-favoring and 50 g/L as titer-maximizing under the tested conditions; neither is presented as a globally optimized precursor concentration. Biomass production decreased as the concentration of L-glutamic acid increased, and little changes in cell growth were observed for cells up to 30 g/L. This result suggests that moderate levels of L-glutamic acid may be sufficient to have enough precursor availability but still have a good physiological state for cell growth. However, excessive supplementation may adversely affect cell growth.

GABA production was in different order from cell growth. At 10 g/L L-glutamic acid strain FBL-3 produced 8.13 ± 2.61 g/L GABA and the concentration of GABA decreased to 5.79 ± 2.09 g/L at 20 g/L. After this decrease, GABA production increased in a concentration-dependent manner as L-glutamic acid was increased. The highest concentration of GABA (19.6 ± 1.03 g/L) was found at 50 g/L for L-glutamic acid. At 50 g/L supplemented L-glutamic acid, the endpoint GABA concentration of 19.6 ± 1.03 g/L corresponded to an average volumetric productivity of 0.408 g/L/h over 48 h and an apparent molar conversion of 56.0%. Because only the endpoint was measured, this value represents average rather than maximum productivity. The conversion estimate is based on supplemented precursor and should be interpreted cautiously in the complex medium. Although the presence of GABA increases with the level of precursor, the magnitude of increase between 40 and 50 g/L is smaller than the increase between 30 and 40 g/L so that the stimulatory effect of L-glutamic acid could have been lower at higher levels.

The significance of strain FBL-3 lies in its origin from godeulppaegi kimchi and in its experimentally defined responses to carbon source, nitrogen source, and L-glutamic acid concentration under a single batch-culture protocol, rather than in an assertion that GABA production is unique to this species. Direct numerical comparison with published *Lvb. brevis* strains must be interpreted cautiously because medium composition, precursor form and concentration, pH strategy, and cultivation time differ among studies (Table 3). Under the highest-titer condition tested here, FBL-3 produced 19.6 ± 1.03 g/L GABA after 48 h, corresponding to an average volumetric productivity of 0.41 g/L/h and an apparent molar conversion of 55.9% relative to the 50 g/L L-glutamic acid supplied. These values place FBL-3 below highly optimized or high-substrate processes, but demonstrate measurable GABA production by a newly isolated food-derived strain under an uncontrolled batch process.

Both biomass formation and accumulation of GABA might be due to the fact that L-glutamic acid is a biosynthetic precursor and substrate for acid stress response. In lactic acid bacteria, glutamate decarboxylase is a protein pathway that converts glutamate into GABA and the antiporter GadC is responsible for the exchange of extracellular glutamate and intracellular GABA [8-10]. This proton-consuming and pH-dependent antiport mechanism is one of the main acid-resistant strategies of Gram-positive fermentative bacteria, where the extracellular glutamate and intracellular GABA are related to the pH gradient in the transmembrane and not to the glutamate concentration alone. A higher concentration of L-glutamic acid provides more substrate for GABA production but an excessive amount of precursor may also reduce cell growth and conversion efficiency by altering osmotic balance, medium pH, and overall physiological state. Prior studies also found that precursor concentration and culture pH should be optimized together, because an excessive glutamate supply also does not always result in better conversion yield even if the final GABA titer is higher [14, 22, 23].

Therefore, the availability of L-glutamic acid is very necessary for the GABA biosynthesis by *Lvb. brevis* FBL-3. Since GABA is produced by decarboxylation of L-glutamic acid by the glutamate decarboxylase system, increasing the precursor supply can be beneficial for substrate availability for the GABA production process. However, the decrease of cell growth at increased L-glutamic acid concentrations suggests that excessive precursor supplementation may affect physiological activity. This could be due to osmotic stress, changes in the medium composition or rapid acidification during fermentation. In particular, the organic acids that are produced during the LAB growth may reduce the pH of the medium and this acidification may limit the cell growth even if precursor availability is high. The different trends of biomass formation and GABA accumulation indicate that the proper L-glutamic acid level is dependent on the evaluation criteria. L-Glutamic acid at 10 g/L is the one that supports the highest cell growth and produced a large amount of GABA with low precursor input. On the other hand, L-glutamic acid that is 50 g/L was found to have the highest final GABA concentration. Thus, 10 g/L L-glutamic acid can be considered an efficient concentration from the cell growth and precursor usage point of view, while 50 g/L is more favorable for final GABA accumulation.

In general, L-glutamic acid was found to be an important precursor of GABA biosynthesis in *Lvb. brevis* FBL-3. Our results also point out that further improvement in GABA production can be achieved by controlling culture pH during fermentation, as pH-controlled or substrate-feeding fermentation has been shown to significantly increase GABA titers in other *Lvb. brevis* strains. Culture pH is worth considering when interpreting the results of this one-factor-at-a-time screening. Because glutamate decarboxylase activity and GadC-mediated glutamate/GABA exchange are tied to acid stress and proton consumption, the pH trajectory during fermentation is relevant here: cultures started at pH 6.2 and, without active control, acidified to pH 4.5 by 48 h. This final pH falls within the range generally favorable for GAD activity, but since pH was not regulated in real time, the shifts occurring over the course of cultivation may have contributed – alongside the carbon, nitrogen, and precursor variables tested – to the biomass and GABA yields observed. Confirming the true optimum will likely require continuous pH monitoring and a tightly controlled-pH process in future work.

In summary, strain FBL-3 was isolated from godeulppaegi kimchi and identified as *Lvb. brevis* by phenotypic, biochemical, and 16S rRNA gene analyses. Sequential one-factor-at-a-time screening showed that xylose, yeast extract, and L-glutamic acid concentration influenced biomass formation and TLC-densitometrically estimated GABA accumulation. Xylose at 10 g/L gave the highest GABA concentration in the xylose-concentration experiment. Yeast extract at 20 g/L was selected as a practical working level because it produced a mean GABA concentration comparable to 50 g/L with less ingredient input, while 50 g/L gave the highest mean titer. L-Glutamic acid at 10 g/L favored biomass, whereas 50 g/L maximized the endpoint GABA titer (19.6 ± 1.03 g/L; average productivity 0.408 g/L/h; apparent molar conversion 56.0%). The sequential one-factor-at-a-time approach identifies favorable conditions within each individual experiment but does not quantify interactions among xylose, yeast extract, L-glutamic acid, and culture pH. Therefore, the selected conditions should not be interpreted as a globally optimized medium. A factorial or response-surface design, followed by an independent validation culture, is required to establish a statistically validated process optimum. These findings provide preliminary condition-screening data for FBL-3 but do not constitute a multivariable process optimum. Furthermore, food origin does not establish strain safety or food-application performance. Genome-based safety assessment, antimicrobial-resistance and biogenic-amine evaluation, HPLC/UPLC confirmation of GABA, controlled-pH multivariable optimization, and validation in an actual food matrix are required before FBL-3 can be considered for use in GABA-enriched fermented foods.

## Figures and Tables

**Fig. 1:**
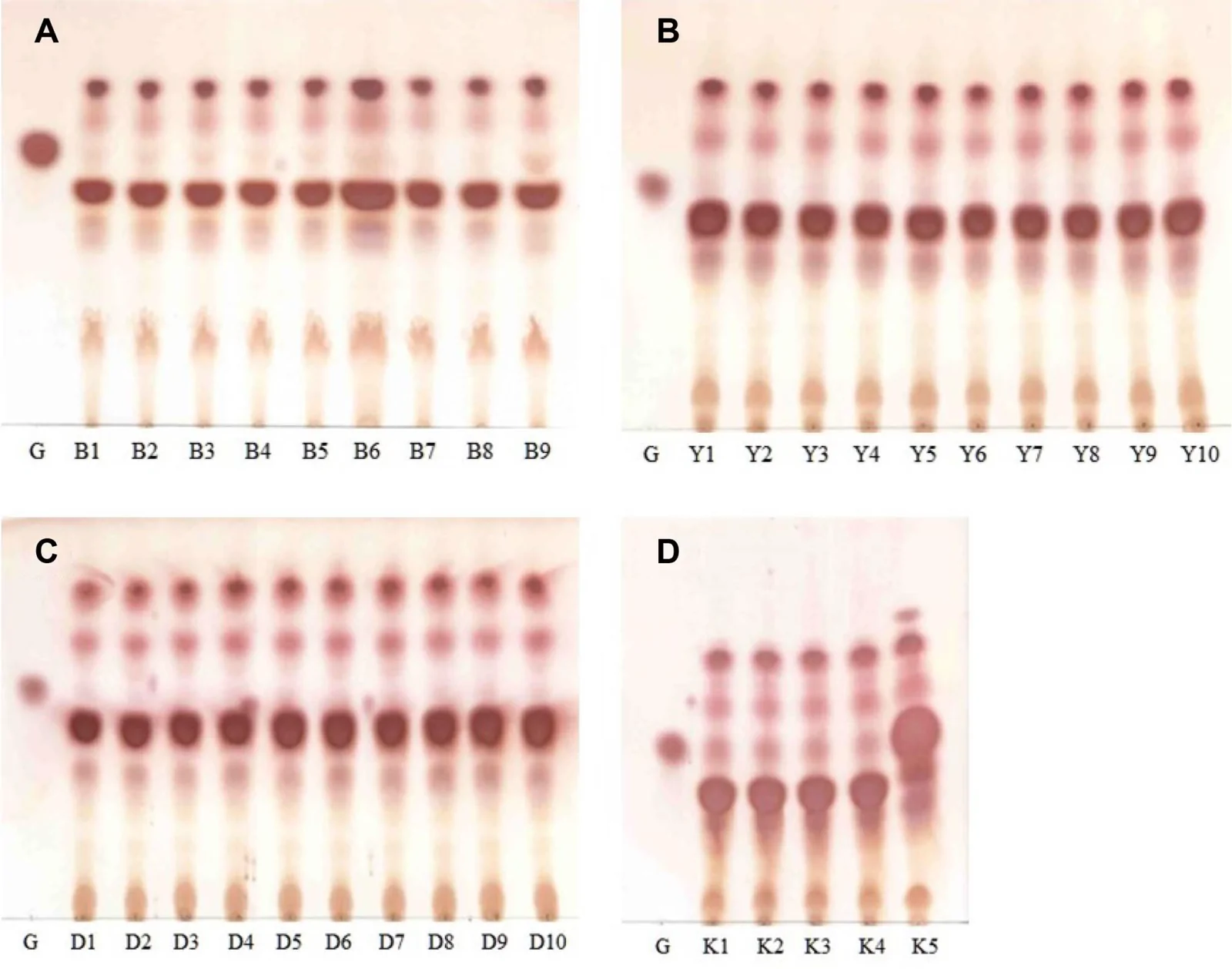
Screening of GABA-producing lactic acid bacteria isolated from different kimchi samples. TLC analysis was performed to compare GABA production by LAB isolates obtained from cabbage kimchi (**A**), young radish kimchi (**B**), dongchimi (**C**), and godeulppaegi kimchi (**D**). Lane G, GABA standard; lanes B1-B9, isolates from cabbage kimchi; lanes Y1-Y10, isolates from young radish kimchi; lanes D1-D10, isolates from dongchimi; lanes K1-K5, isolates from godeulppaegi kimchi.

**Fig. 2:**
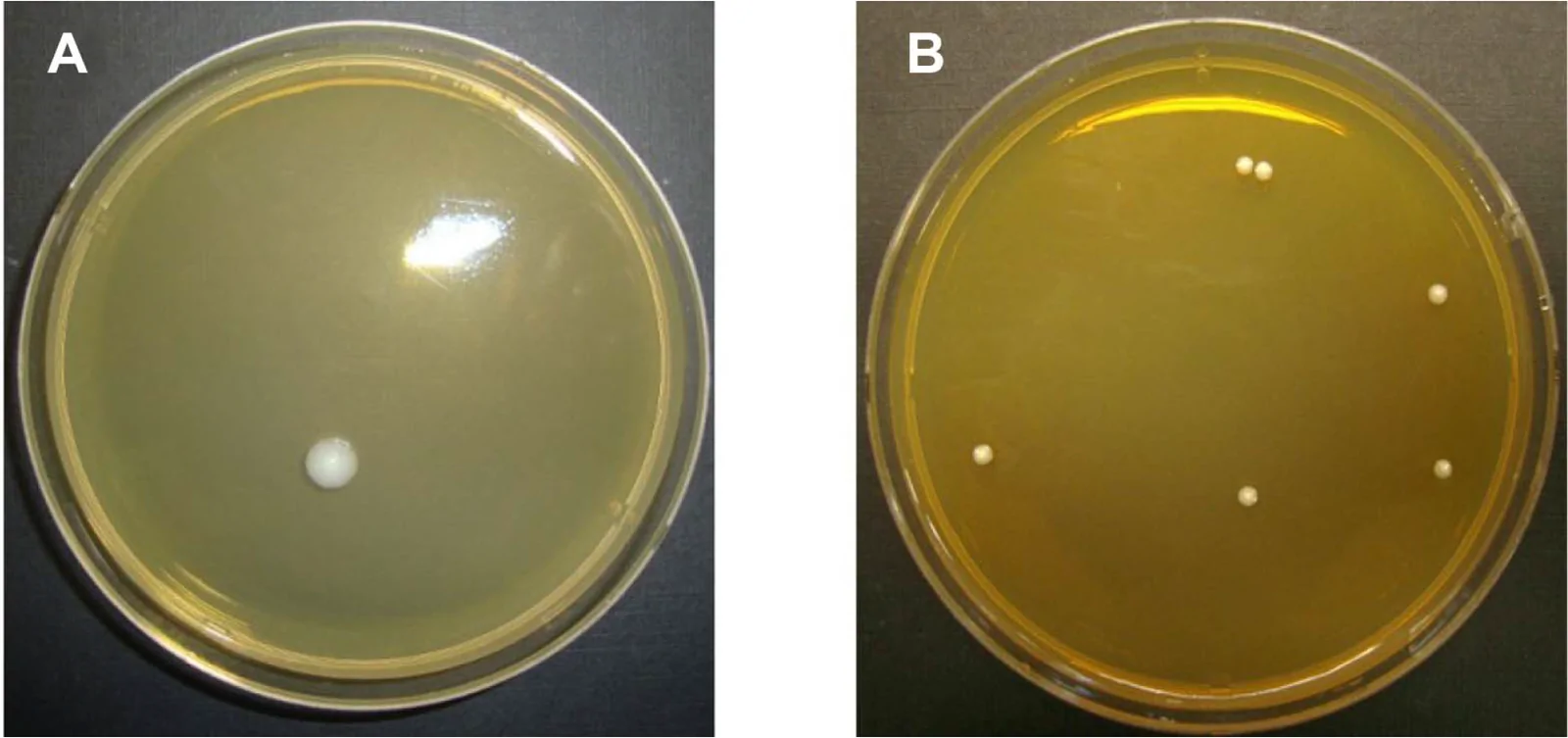
Colony morphology of strain FBL-3. Strains were grown on MRS agar (**A**) and GABA-production agar (**B**) medium.

**Fig. 3:**
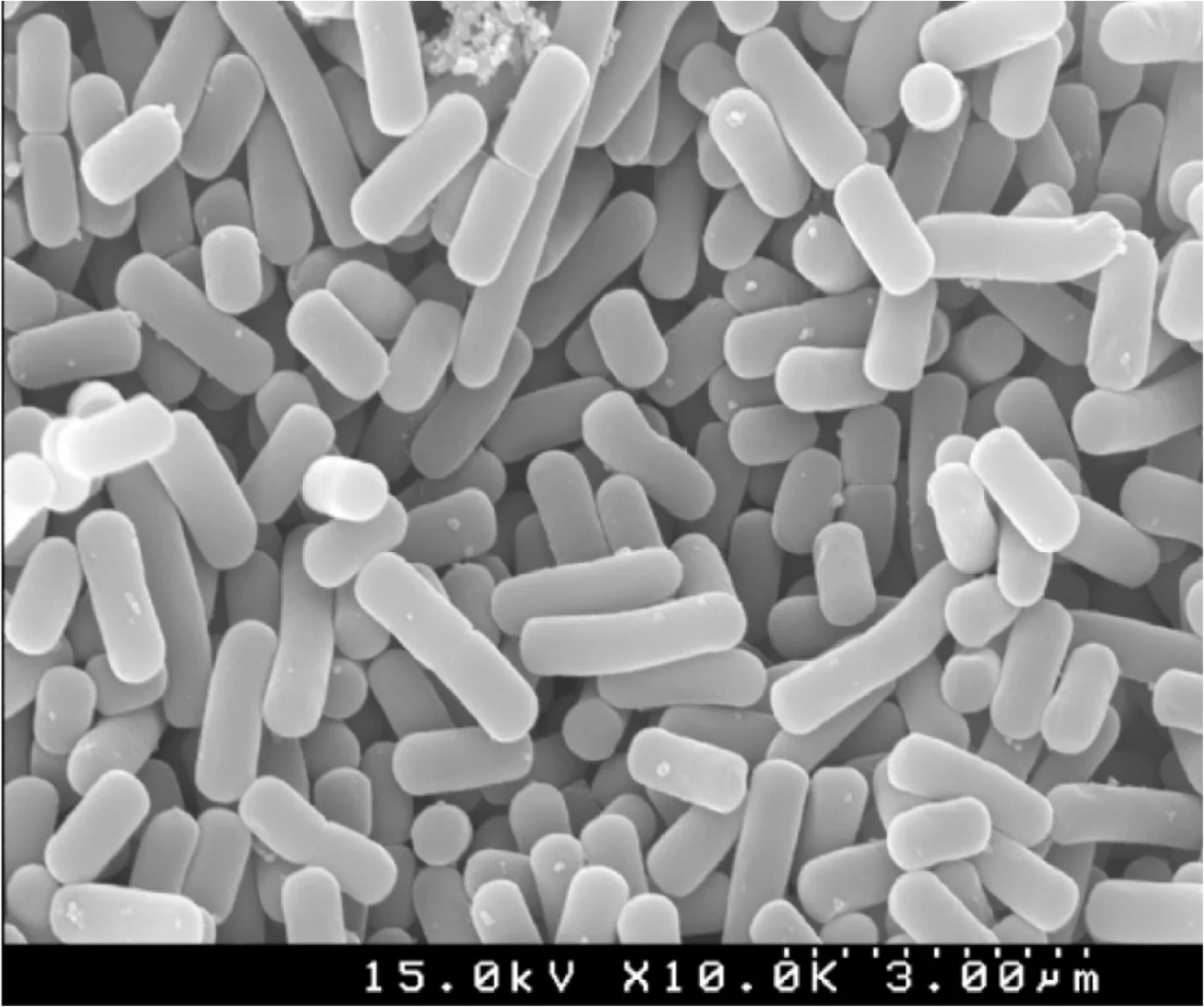
Scanning electron micrograph of strain FBL-3.

**Fig. 4:**
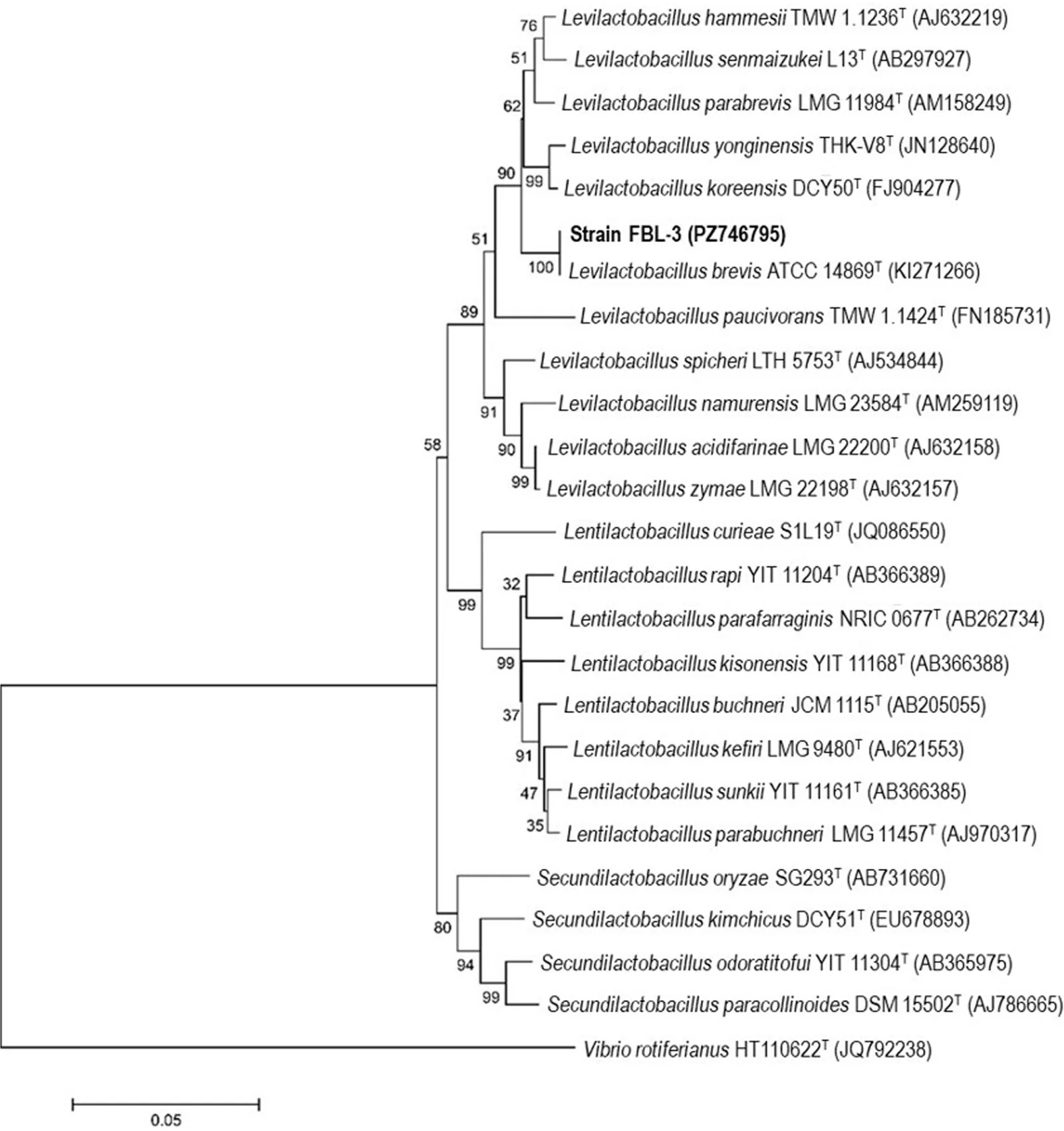
Phylogenetic tree based on 16S rRNA gene sequences showing the evolutionary relationship between strain FBL-3 and other related taxa. The tree was constructed by the neighbor-joining method. *Vibrio rotiferianus* HT110622 was used as outgroup in the tree. Numbers at nodes are bootstrap values expressed as percentages of 1,000 replications. GenBank accession numbers of each sequence are indicated in parentheses. Bar represents 5 substitutions per 100 nucleotides. The superscript ^'T'^ indicates type strain.

**Fig. 5 F5:**
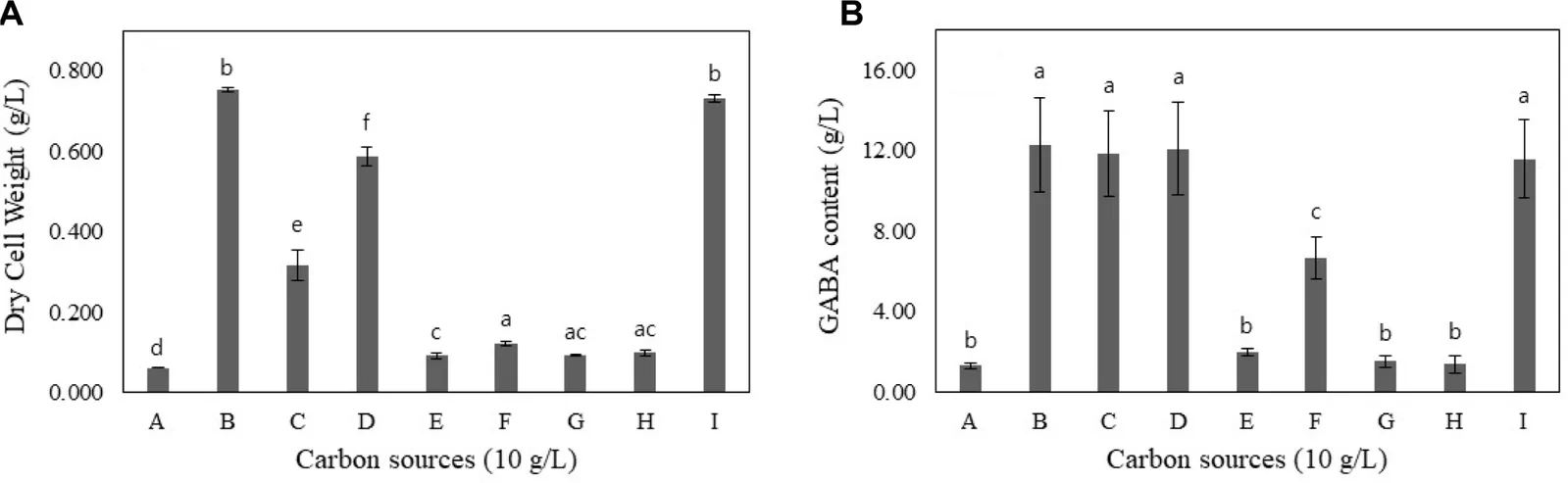
The effect of carbon sources on cell growth (A) and GABA production (B) by *Lvb. brevis* FBL-3. a, none; b, xylose; c, glucose; d, fructose; e, starch; f, galactose; g, sucrose; h, lactose; l, maltose.

**Fig. 6:**
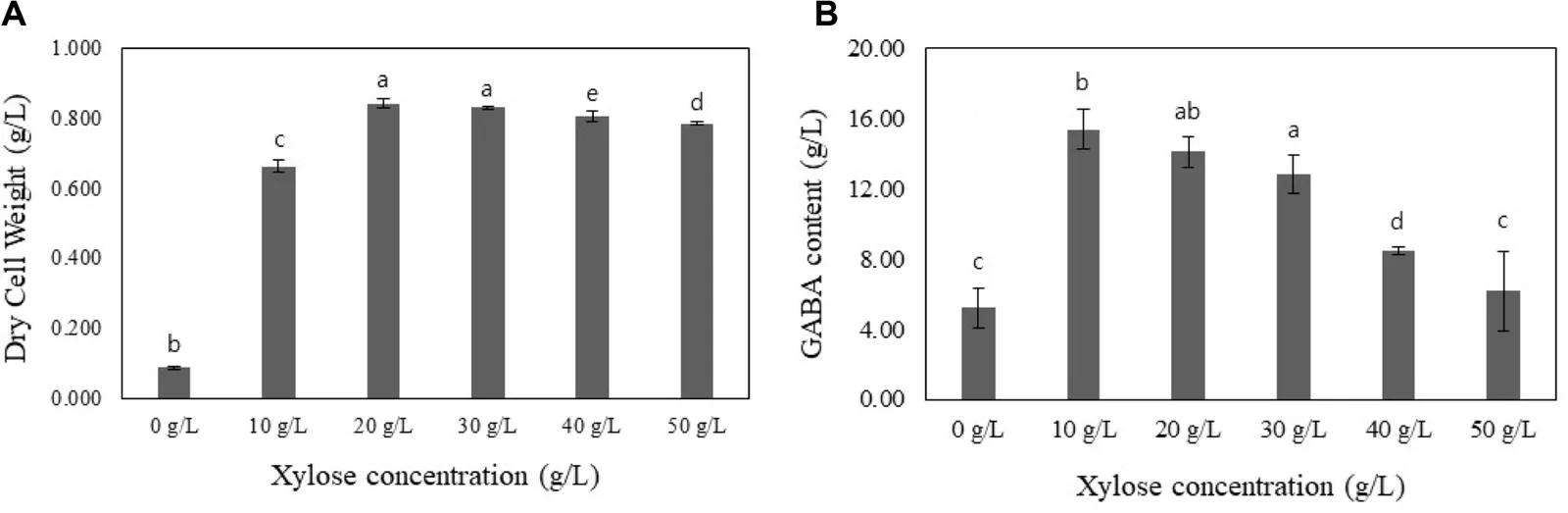
The effect of xylose concentrations on cell growth (A) and GABA production (B).

**Fig. 7:**
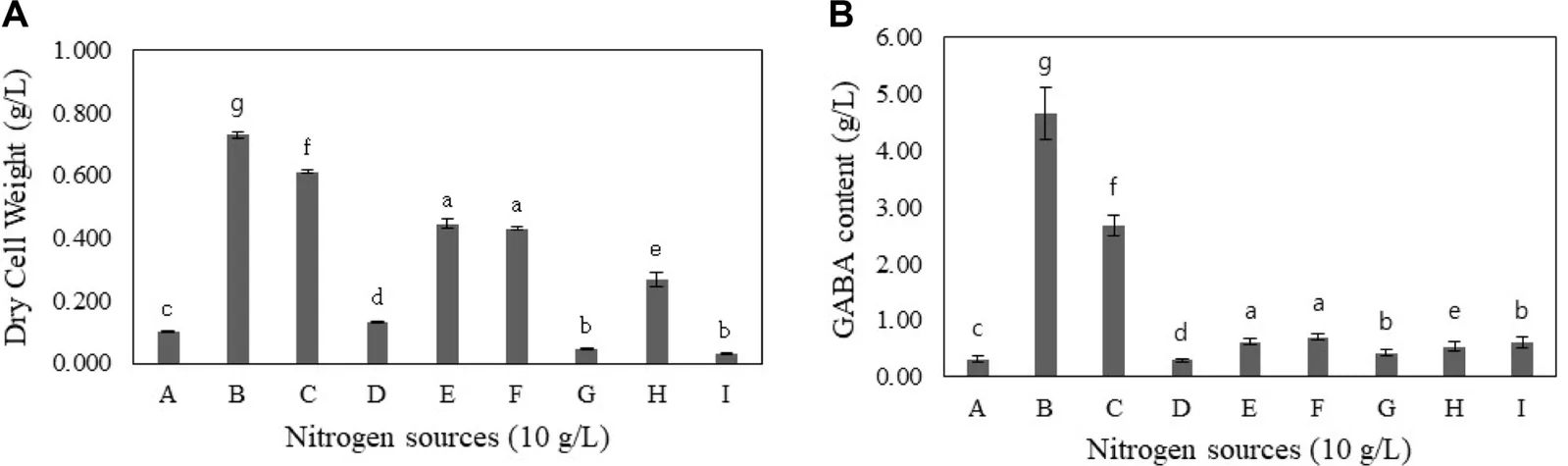
The effect of nitrogen sources on cell growth (A) and GABA production (B) by *Lvb. brevis* FBL-3. a, none; b, yeast extract; c, beef extract; d, malt extract; e, peptone; f, tryptone; g, urea; h, corn steep liquor; i, ammonium chloride.

**Fig. 8:**
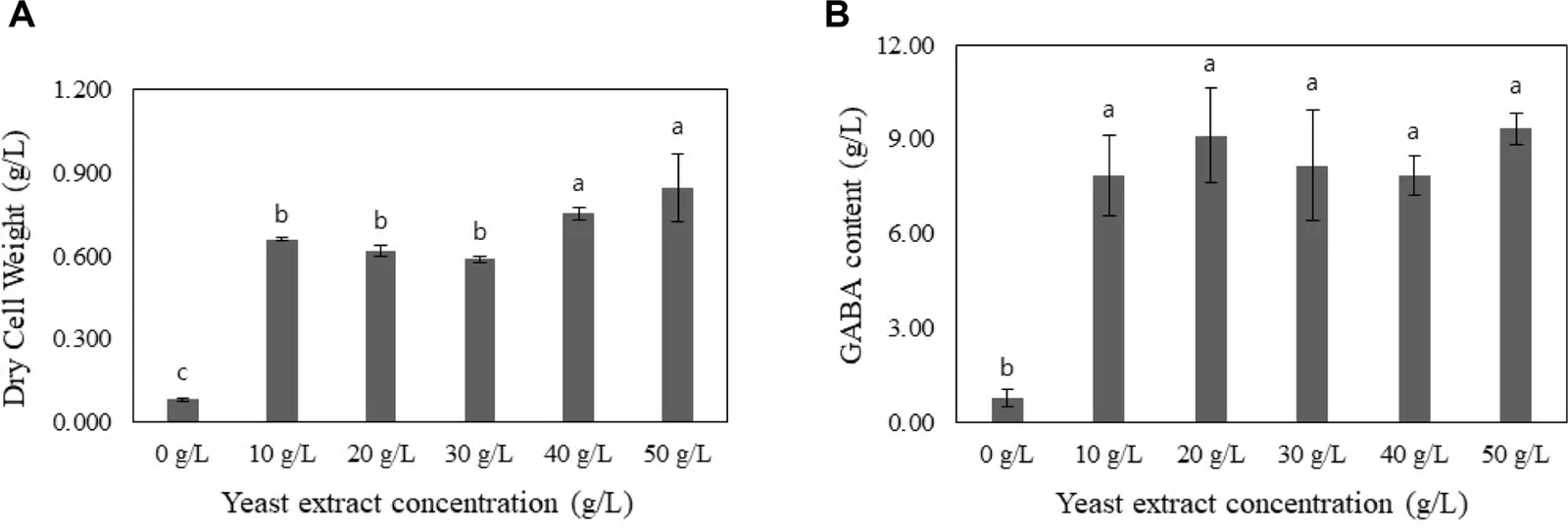
The effect of yeast extract concentrations on cell growth (A) and GABA production (B).

**Fig. 9:**
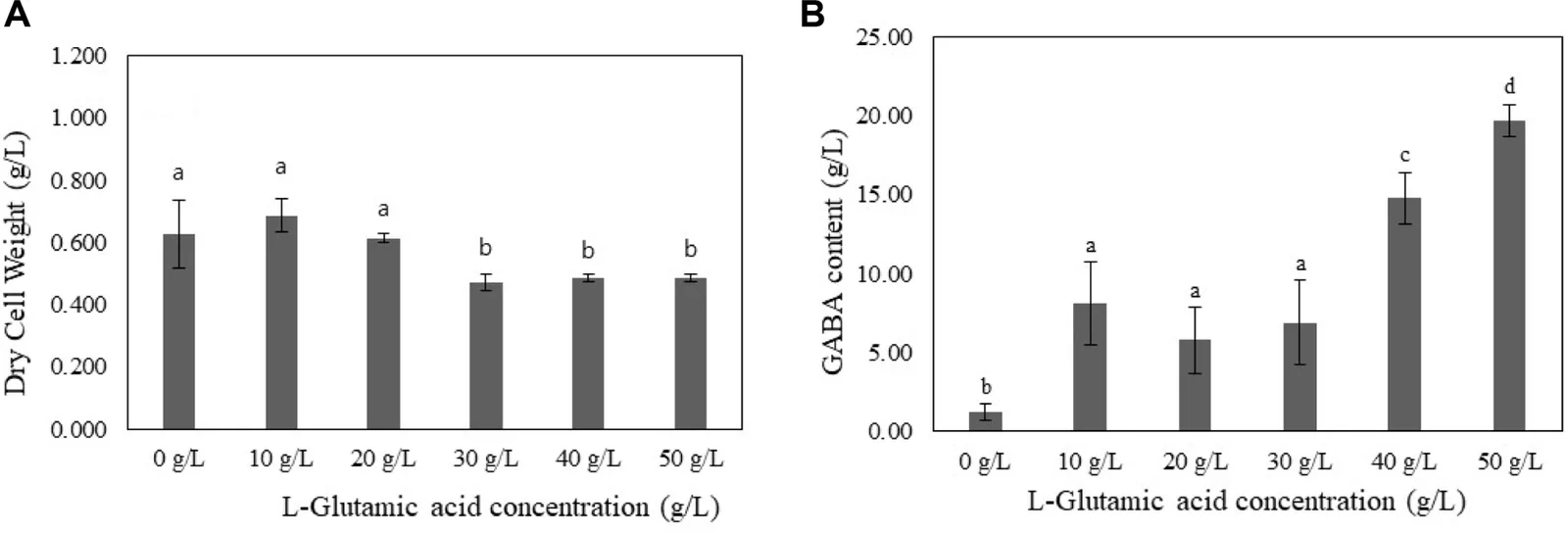
The effect of L-glutamic acid concentrations on cell growth (A) and GABA production (B).

**Table 1:** Phenotypic diversity of strain FBL-3 and related strain of *Levilactobacillus brevis*.

Characteristics	Strain FBL-3	*Lvb. brevis* KCCM 40399	*Lvb. brevis* KCTC 3498
Utilization of (with API 50CHL kit)			
Control	-	-	-
Glycerol	-	-	-
Erythritol	-	-	-
D-Arabinose	-	-	-
L-Arabinose	+	+	+
Ribose	+	+	+
D-Xylose	+	+	+
L-Xylose	-	-	-
Adonitol	-	-	-
β-Methyl-D-xyloside	-	-	-
D-Galactose	-	+	-
D-Glucose	-	+	-
D-Fructose	+	+	?
D-Mannose	-	-	+
L-Sorbose	-	-	-
Rhamnose	-	-	-
Dulcitol	-	-	-
Inositol	-	-	-
Mannitol	-	-	-
Sorbitol	-	-	-
α-Methyl-D-mannoside	-	-	-
α-Methyl-D-glucoside	-	-	-
N-Acetyl-glucosamine	-	+	?
Amygdalin	-	-	-
Arbutin	-	-	-
Esculin	-	-	-
Salicin	-	-	-
Cellobiose	-	-	-
Maltose	+	+	+
Lactose	-	-	-
Melibiose	-	-	-
Sucrose	-	-	-
Trehalose	-	-	-
Inulin	-	-	-
Melezitose	-	-	-
Raffinose	-	-	-
Starch	-	-	-
Glycogen	-	-	-
Xylitol	-	-	-
Gentiobiose	-	-	-
D-Turanose	-	-	-
D-Lyxose	-	-	-
D-Tagatose	-	-	-
D-Fucose	-	-	-
L-Fucose	-	-	-
D-Arabitol	-	-	-
L-Arabitol	-	-	-
Gluconate	-	+	?
2-Keto-gluconate	-	-	-
5-Keto-gluconate	+	-	?

+, positive reaction; -, negative reaction

**Table 2:** Pairwise similarity and nucleotide difference of the related species based on their 16S rRNA gene sequence compared to strain FBL-3.

Strain	Accession number	Pairwise similarity (%)	Nucleotide difference/compared
*Levilactobacillus brevis* ATCC 14869^T^	KI271266	100.00	0/1437
*Levilactobacillus hammesii* TMW 1.1236^T^	AJ632219	98.40	23/1437
*Levilactobacillus yonginensis* THK-V8^T^	JN128640	98.11	27/1431
*Levilactobacillus senmaizukei* L13^T^	AB297927	98.11	27/1426
*Levilactobacillus koreensis* DCY50^T^	FJ904277	97.97	29/1430
*Levilactobacillus spicheri* LTH 5753^T^	AJ534844	97.84	31/1437
*Levilactobacillus parabrevis* LMG 11984^T^	AM158249	97.84	31/1435
*Levilactobacillus acidifarinae* LMG 22200^T^	AJ632158	97.35	38/1434
*Levilactobacillus zymae* LMG 22198^T^	AJ632157	97.21	40/1436
*Levilactobacillus namurensis* LMG 23584^T^	AM259119	96.66	48/1437
*Levilactobacillus paucivorans* TMW 1.1424^T^	FN185731	95.82	60/1437
*Secundilactobacillus kimchicus* DCY51^T^	EU678893	94.84	74/1435
*Secundilactobacillus oryzae* SG293^T^	AB731660	94.70	76/1435
*Lentilactobacillus buchneri* JCM 1115^T^	AB205055	94.64	77/1437
*Lentilactobacillus rapi* YIT 11204^T^	AB366389	94.64	77/1437
*Lentilactobacillus parabuchneri* LMG 11457^T^	AJ970317	94.49	79/1435
*Lentilactobacillus kisonensis* YIT 11168^T^	AB366388	94.43	80/1437
*Secundilactobacillus odoratitofui* YIT 11304^T^	AB365975	94.39	80/1426
*Lentilactobacillus sunkii* YIT 11161^T^	AB366385	94.36	81/1437
*Lentilactobacillus curieae* S1L19^T^	JQ086550	94.36	81/1437
*Lentilactobacillus kefiri* LMG 9480^T^	AJ621553	94.35	81/1433
*Lentilactobacillus parafarraginis* NRIC 0677^T^	AB262734	94.29	82/1437
*Secundilactobacillus paracollinoides* DSM 15502^T^	AJ786665	94.22	83/1435

^T^type strain

**Table 3:** Comparison of GABA production by representative non-engineered *Levilactobacillus brevis* strains.

Strain	Origin	Representative culture conditions	Initial glutamate/MSG	Time (h)	Final GABA (g/L)	Average productivity (g/L/h)	Molar conversion (%)
*Lvb. brevis* FBL-3 (this study)	Godeulppaegi kimchi (Korean bitter lettuce kimchi)	Xylose 10 g/L, yeast extract 20 g/L, K_2_HPO_4_ 1 g/L, L-glutamic acid 50 g/L; initial pH 6.2; 37°C; anaerobic; 200 rpm	50 g/L L-glutamic acid (339.8 mM)	48	19.6 ± 1.03	0.41^†^	55.9^a,b^
*Lvb. brevis* HYE1 [9]	Kimchi	Maltose 2.14% (w/v), tryptone 4.01% (w/v), MSG 2.38% (w/v); initial pH 4.74; 30°C	2.38% (w/v) MSG	48	18.76 mM (1.93 g/L)	0.040	13.3
*Lvb. brevis* K203 [11]	Kimchi	L-glutamic acid 6% (w/v), maltose 4% (w/v), yeast extract 2% (w/v), NaCl 1% (w/v), CaCl_2_ 1% (w/v), Tween 80 2 g/L, PLP 0.02 mM; initial pH 5.25; 37°C	6% (w/v) L-glutamic acid (407.8 mM)	72	44.4 g/L	0.62	99.7
*Lvb. brevis* CRL 1942 [12]	Real Hornillos quinoa sourdough	MRS broth supplemented with 270 mM MSG; capped test tubes; no shaking; 30°C	270 mM MSG	48	~255 mM (~26.3 g/L)	-0.55	94.4

Values are compared descriptively because media composition, pH strategy, analytical method, and culture scale differed among studies.

^a^Calculated as final GABA titer divided by fermentation time, or from the stated initial precursor concentration and molecular weights of GABA (103.12 g/mol) and L-glutamic acid (147.13 g/mol).

^b^The value for FBL-3 is an apparent molar conversion based on supplemented L-glutamic acid; complex medium ingredients and inoculum carryover may contribute additional glutamate, and residual substrate was not used for this calculation.
